# Oxidizability assay of unfractionated plasma of patients’ with different plasma profile: a methodological study

**DOI:** 10.1186/2251-6581-13-54

**Published:** 2014-05-01

**Authors:** Hasan Imam, Arfia Chowdhury, Nasir Uddin Mahbub, Amir Hossain, Mohammed Faisal Bin Karim, Mohammad Burhan Uddin, Md Moklesur Rahman Sarker

**Affiliations:** 1 Department of Pharmacy, Primeasia University, 9 Banani C/A, Dhaka 1213, Bangladesh; 2 Exim Bank Hospital, 840 Rokeya Soroni, Mirpur, Dhaka 1216, Bangladesh; 3 Clinical Investigation Centre, Faculty of Medicine, University of Malaya, 50603 Kuala Lumpur, Malaysia

**Keywords:** Plasma oxidation, Conjugated diene, TBARS, Type II Diabetes

## Abstract

**Background:**

Present study describe the in vitro model of plasma oxidation of patients with different lipid profile, that can be correlated to their invivo plasma oxidizability in order to find the arterial diseases prone patient groups.

**Method:**

The method applied here to measure the invitro plasma oxidizability, accounts a convenient way that can be well suited in any clinical laboratory settings. Un-fractionated plasma was exposed to CuSO4 (5.0 mmol/L), a pro-oxidant, and low frequency ultrasonic wave to induce oxidation, and finally oxidizability was calculated by TBARS and Conjugated Diene methods.

**Result:**

In our study, plasma LDL greater than 150 mg/dL possess 1.75 times more risk to undergo oxidation (CI, 0.7774 to 3.94; p = 0.071) than the low LDL plasma, percent of oxidation increased from 38.3% to 67.1% for the LDL level upto 150 mg/dL and high. Lag phase, which is considered as the plasma antioxidative protection, was also influenced by the higher LDL concentration. The mean lag time was 65.27 ± 20.02 (p = 0.02 compared to healthy), where as for 94.71 ± 35.11 min for the normolipidemic subject. The plasma oxidizability was also changed drastically for total cholesterol level, oxidative susceptibility shown 35% and 55.02% for 200 mg/dL and high respectively, however it didn’t appear as risk factor. Patient samples were also stratified according to their age, gender, and blood glucose level. Older persons (≥40 years) were 1.096 times (95% CL, 0.5607 to 2.141, p = 0.396) than younger (≤39 years age), males are 1.071 (95% CI, 0.5072- 2.264) times than the females, and diabetic patients are 1.091 (CI, 0.6153 to 1.934, p = 0.391) times in more risk than the non-diabetic counterpart.

**Conclusion:**

This method addressing its easy applicability in biomedical research. And by this we were able to show that patients with high LDL (≥150 mg/dL) are in alarming condition besides diabetic and elderly (≥40 years age) males are considered to be susceptible and more prone to develop vascular diseases.

## Introduction

The clinical importance of lipid oxidation, mainly low density lipoproteins (LDLs) is presumably associated with atherosclerosis formation [1]. Though in biological model it is not easy to show the pathological process of atherosclerosis, however the in vitro oxidizability of patients’ plasma could be extrapolated to assess the susceptible patient group [2]. Many studies has shown the Cu^2+^ mediated oxidation of isolated LDL and fatty acids [2-4], even in unfractionated plasma [2]. We have exercised here also the same protocol to study the unfractionated plasma in the presence of CuSO4, but there was not enough differences observed in plasma lipid oxidation between presence and absence of CuSO4 alone. We made a slight modification by exposing them in ultrasonic wave so that the lipid portion in the solution would be favorable in size to undergo the oxidative modification.

Several epidemiological investigations implicate the relationship between high cholesterol level with new onset of coronary heart disease (CHD) [5-8] in patients who were not previously affected, and newly appearance of coronary artery disease in recognized CHD patients [9-11]. From their results it appeared that the level above 100 mg/dL of LDL cholesterol is atherogenic. In contrast, it also shown that people with low level of cholesterol are less prone to develop atherosclerosis and CHD [12-14]. So there is a positive relationship observed between serum cholesterol level and CHD. But the threshold level for this relationship is not very much straightforward. Again some populations were free from CHD by maintaining below 150 the total cholesterol or below 100 mg/dL LDL cholesterol level [15-18]. In case of higher cholesterol level with young adults were found increased chances to develop CHD in later stage of their life [19,20]. On the other hand, low HDL level also posses the risk of developing CHD, though it holds the independent relationship. In genetically modified animal model, it appears that the higher level of HDL protects it against atherogenesis [21-23] and deficiency of HDL exacerbates it [24,25]. Moreover the antioxidant and anti-inflammatory effect of HDL can also impart the anti-atherogenic role [26,27]. So, there is a lot of published work to support the role of lipoproteins on oxidative stress but the oxidative susceptibility status with different types of lipoproteins and cholesterol levels in patient’s plasma has not been studied in detail.

To address the susceptibility value of patients’ unfractionated plasma lipid in terms of their plasma lipid profile as a prototype for the ex vivo lipid oxidation, we have developed a very convenient and less equipped model to assess data in large scale. We hypothesized that plasma lipid oxidizability would be sensitive with the following parameters: level of lipoproteins, cholesterol, triglycerides, age, diabetes mellitus and sex. In order to test this hypothesis, we carried out three studies- firstly we examined the plasma lipid profile and then in vitro plasma lipid oxidation following conjugation diene formation and TBARS formation, in the presence of Cu^++^ ion with occasional exposure of ultrasonic wave.

## Methods

### Sample collection

Study subjects included 45 male and female (35 – 75 years old). Among them, 19 were with LDL level was higher than 100 mg/dL, 14 individuals with greater than 150 mg/dL level of total cholesterol, 18 individuals with lower than 35 mg/dL of HDL, number of young patients (age below 40 years) were 23 and older (above 40 years) 22, whereas patients with diabetes mellitus were 15. Peripheral venous blood samples of all patients’ were collected on heparin (5 U/ml) from subjects with 12 hours fasting. Plasma was separated by 5 min centrifugation at 4000 rpm immediately after collection. Data acquisition were completed on the same day, alternatively, the samples were stored at 4°C for 24 hours or at -20°C for not more than 2 days. Informed consent form was made and approved according to the recommendation of the University Medical Research Ethical Committee of the Faculty of Science, Primeasia University, Dhaka, Bangladesh, where the objective of this study and use of biological samples were clarified.

### Biochemical analysis

Blood glucose, total cholesterol (TC), triglyceride (TG) and HDL level were estimated following the standard laboratory methods using 3000-Evolution analyzer (Italy). And Friedewald’s formula [28] was applied for LDL estimation.

### Plasma oxidizability measurement following thiobarbituric acid reactive substance formation (TBARS) method

TBARS formation was measured following the method described by Adriana E Scoccia [3], malonaldihyde, a secondary metabolite and a marker of lipid peroxidation and oxidative stress, which reacts with Thiobarbituric Acid (TBA) to yield pink colored complex that can be measured spectophotometrically. Briefly, 50 μL plasma was diluted in 10 mL CuSO4 (5.0 mmol/L)solution and incubated at 37°C for 3 hours in ultrasonic waterbath with occasional exposure of ultrasonic wave (SonoSWISS SW6 H, Switzerland) to allow the oxidation process. Then 0.5 mL of 0.78% aqueous solution of thiobarbituric acid and 50 μL of acetic acid was added in 0.5 mL of the plasma-CuSO4 mixture. Control was prepared in the same way except CuSO4. The mixture was heated at 80°C for 40 min in a ultrasonic waterbath and absorbances were taken at 532 nm using a spectrophotometer (SHIMADZU UV 1650 pc, UV–vis. Spectrophotometer, Japan). Percentage of oxidation was calculated as follows-

$$\mathrm{Oxidation}\left(\%\right)=\left(\mathrm{Aps}-\mathrm{Ac}\right).100/\mathrm{Ac}$$

Where *Aps* is the absorbance of plasma sample, *Ac* is absorbance of control.

### Plasma oxidizability measurement following conjugated diene method

Conjugated diene was measured following a slight modification of the method described by Kontush and Beisiegel [29], where photometrical detection is employed for the conjugated diene. In brief, 50 μL plasma was diluted to 10 mL phosphate-buffered saline, to which CuSO4 was added to make the concentration 5.0 mmol/L. In another tube, 5.0 mmol/L CuSO4 in phosphate-buffered saline was served as a blank. Samples, in cuvettes, after a brief exposure of ultrasonic wave were incubated in spectrophotometer (SHIMADZU UV 1650 pc, UV–vis. Spectrophotometer, Japan) for 160 min, where sample absorbance was measured at 234 nm in every 2 min interval. Lag phase was considered for the time taken with no increment of absorbance before a rapid oxidation. The duration of lag phase indicate the resistance to oxidation. CD_max_ was calculated from the maximum absorbance value and the Beer-Lambert law, where the extinction coefficient for conjugated diene is 29500 L.mol/cm [4].

### Statistical analysis

Results are presented as interquartile ranges or as means ± SDs. Statistical analysis between normal and test group was performed using Microsoft Excel 2007 and data were compared with unpaired two tailed *t*-test. Risk ratio within 95% confidence interval was calculated using Openepi_37V software (http://www.openepi.com). Mid p-exact was taken, where p value threshold was used ≤0.05 to indicate a statistical significant.

## Result and discussion

**Figure 1:**
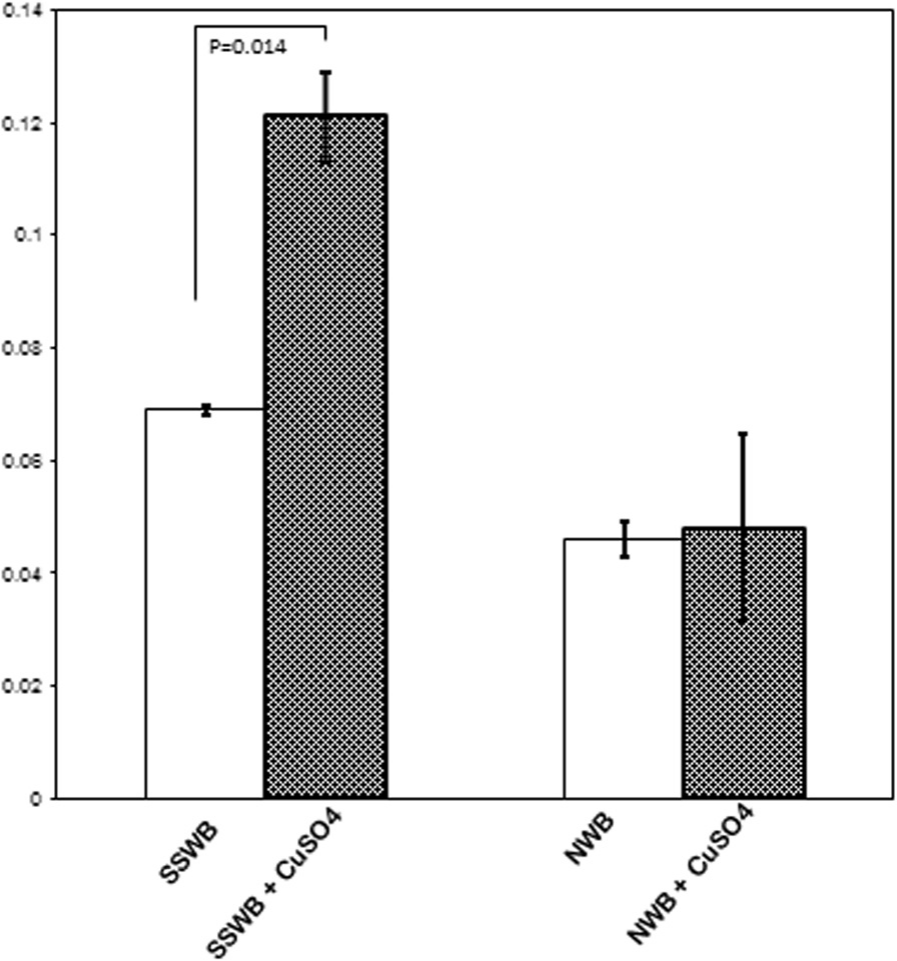
**Comparative oxidation of unfractionated plasma by incubating in two different conditions at 37°C, here SSWB means Sono Swiss water bath with ultrasonic wave exposure, and NWB means normal water bath.** Bars are presented; mean (absorbance) ± SD. Difference in oxidation of plasma between presence and absence of Cu^2+^ in ultrasonic bath was significant.

**Figure 2:**
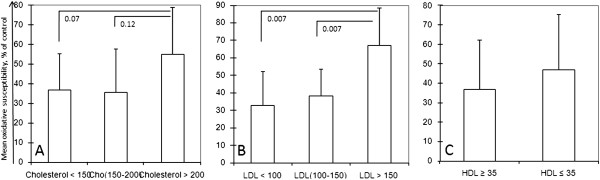
**Plasma oxidizability with different plasma profile. (A)** Plasma oxidizability in terms of total cholesterol level. **(B)** Plasma oxidizability in terms of different LDL concentration, which was significantly higher in plasma with highest LDL level than intermediate level (p = 0.0068) and lower level (p = 0.007). **(C)** Plasma oxidizability of different samples with different HDL level. Oxidizability is shown by mean (percent of basal) ± SD.

In this in vitro Cu^++^ and ultrasonic wave mediated oxidative susceptibility assay, first we compared the TBARS level by incubating tube containing patients’ plasma with CuSO4 at 37°C in normal waterbath for 3 hours. Having not any comparable data between treated and untreated sample, we slightly modified the method by exposing ultrasonic wave during this incubation period and a comparable data was generated (Figure 1). This modified method for TBARS assay was exercised throughout the experiment. We observed the percent oxidizability in terms of plasma cholesterol level (Figure 2A) for the first two subgroups were fairly similar, 36.95% and 35.60% respectively for plasma below 150 and 150-200 mg/dL, but it jumped to 55.02% for the patients with higher than 200 mg/dL, although not significantly (p = 0.124) compared to the oxidative susceptibility of 150 mg/dL. However, the risk ratio appeared not so high, 1.003.

Similar trend was observed comparing the plasma LDL level, which appeared as 32.70 and 38.30% in case of 100 mg/dL and 100-150 mg/dL subgroup, respectively compared to the control. But it rose as high as 67.18 percent for LDL level ≥150 mg/dL, which was statistically significant compared to the other two subgroups (Figure 2B. p-values were 0.007 for both cases). This result was supported by the findings of other group’s reporting [3], where TBARS formation was linearly increased with increasing volume of LDL. And the risk ratio demonstrate that higher LDL level indulge patients in a condition which is 1.75 times more likely to occur arterial diseases than the low LDL level; the 95% confidence interval around this estimate is 0.7774, 3.94 (p = 0.071).

In Figure (2C) the bar chart compares the serum oxidative susceptibility in terms of their plasma HDL concentration. The mean susceptibility was 47.04 and 37.05% for the people with less than 35 and greater that 35 mg/dL of HDL level in their serum.

People with diabetes mellitus were shown to possess higher plasma oxidizability by both TBARS and CD_max_ result (Table 1), and are 1.091 time more in chance to develop arterial disease than the non-diabetic group, with a 95% confidence interval of 0.6153 to 1.934.

**Table 1:** Plasma oxidative susceptibility in different traits

**Trait**	**Subgroup**	**N**	**Mean oxidative susceptibility by TBARS**	**CD **_**max **_**(μmol/mL plasma)**	**Relative risk**	**Confidence interval**
**Gender**	Male	21	42.405 ± 21.53	0.0544 ± 0.002	1.071 (in male than females, p = 0.432)	0.5072 to 2.264
	Female	24	43.137 ± 26.95	0.059 ± 0.005		
**Age**	Younger (age below 40 years)	23	29.375 ± 20.13	0.0545 ± 0.0032	1.096 (in older than younger, p = 0.39)	0.5607 to 2.141
	Older (above 40 years)	22	54.336 ± 22.25	0.0575 ± 0.004		
**Diabetes**	No	30	28.375 ± 19.56	0.0565 ± 0.0032	1.091(in non-diabetes than diabetes, p = 0.39)	0.6153 to 1.934
	Yes	15	38.607 ± 18.61	0.0573 ± 0.0053		

An analysis between male and female and between younger (age below 40) and older (above 40) (Table 1), not considering their diabetes status, showing that the risk ratio for males is 1.071 (95%, CI 0.5072- 2.264) and for older is 1.096 (0.5607 to 2.141). But none of these results were statistically significant.

There is not much differences are showing in plasma oxidizability between male and female in terms of their TBARS formation. However, it gave statistically significant difference in conjugated diene formation. Turning to the reasons for the higher value to CD_max_, there were almost half of the women in the presented data were hyperlipidemic. A circuitous role of oxygen free radical formation from polymorphonuclear leukocytes and monocytes in hyperlipidemic subject have previously been described [30].

**Figure 3:**
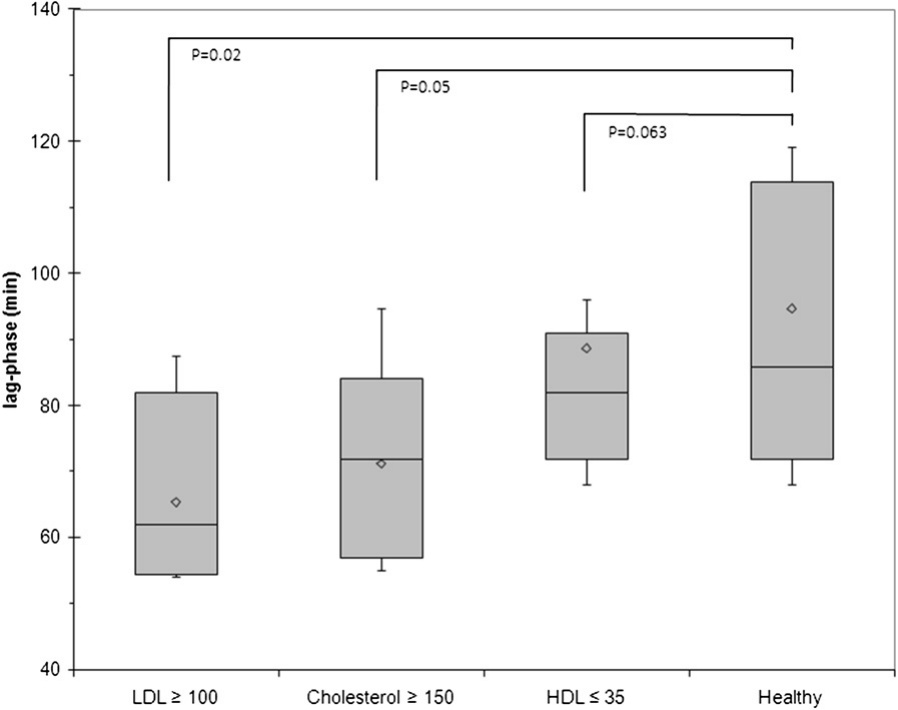
**Box plot of lag-time in conjugated diene assay.** The boxplot shows variable range of Lag time in conjugated diene assay, median values were 86.00, 82.00, 72.00 and 62.00 for healthy, patient group with less than 35mg/dL HDL, greater than 150 mg/dL cholesterol and ≥100 mg/dL LDL level, with average values 94.71, 88.72, 71.25 and 65.27 respectively.

Mean lag phase are declined in patients with high LDL and total plasma cholesterol level (Figure 3). The downward shift, presented in lag phase with LDL and total cholesterol level higher than 100 and 150 mg /dL, are statistically significant (p = 0.02 and 0.05, respectively, compared to healthy volunteer who were normolipidemic). Antioxidant defense, attributed mainly by the lipophilic antioxidant vitamin E, and other vitamins play role in the lag phase, which is increased by the presence of these vitamins [2,31]. Surprisingly, the mean lag phase of patients with low HDL level are shown to possess high values. The upward shift is present at even the higher (75^th^) percentile in case of HDL value ≤35 mg/dL. Thus, the upward shift of lag phase in ≤35 mg/dL HDL level is not solely attributed to the lower amount of HDL present in the plasma but must have resulted from the influence of other parameters.

No doubt the lowering of LDL and total cholesterol should be the primary goal for patients with CHD, but it could be beneficial knowledge for general patients’ if their plasma lipid oxidizability values are explained in a simpler way. Unlike the other methods where fractioning LDL or HDL by ultracentrifugation, dialysis, electrophoresis etc. need much effort. Also the oxidation study with isolated lipoproteins may raise question about it’s in vivo comparability [2]. Again this method offers a easy alternative, and substantial data can be generated with less equipped and simple methodological approach like it which is clinically relevant. The increase in TBARS and reduction in CD indicating the less antioxdative protection and high oxidative stress. Data might be influenced by dietary and drug treatment, since the history were not taken. However, standardized experimental condition including randomization, minimum delaying in data acquisition and triplicate sampling outweigh possible obstacles. We conclude that patients with high LDL (≥150 mg/dL) are in alarming condition besides diabetic patients, and elderly (age above 40 years) males are considered to be susceptible and more prone to develop vascular diseases.

## Competing interests

The authors declare that they have no competing interests.

## Authors’ contributions

Research concept, data analysis & writing: HI; Patient sample and data acquisition: AC NM and AH; Coordination: FBK and MBU; Oversaw the study design and helped in drafting the manuscript: MMRS; Instrumental support: AH. All authors read and approved the final manuscript.
